# Group-velocity-locked vector solitons and dissipative solitons in a single fiber laser with net-anomalous dispersion

**DOI:** 10.1038/s41598-022-10818-4

**Published:** 2022-04-27

**Authors:** Shutao Xu, Ahmet Turnali, Michelle Y. Sander

**Affiliations:** 1 Department of Electrical and Computer Engineering and BU Photonics Center, Boston University, Boston, MA 02215 USA; 2 Division of Materials Science and Engineering, Boston University, Brookline, MA 02446 USA; 3 Department of Biomedical Engineering, Boston University, Boston, MA 02215 USA

**Keywords:** Solitons, Nonlinear optics, Lasers, LEDs and light sources, Fibre lasers, Mode-locked lasers, Ultrafast lasers

## Abstract

Laser cavities which can generate different types of ultrashort pulses are attractive for practical applications and the study of pulse dynamics. Here, we report the first experimental observation of both conventional solitons (CS) and dissipative solitons (DS) generated from a single all-fiber laser with net-anomalous dispersion. A birefringence-related intracavity Lyot filter with an adjustable extinction ratio enables the switching between the two types of ultrashort pulses. Depending on the polarization controller settings and the pump power, either chirp-free CS with a pulse energy of 406 pJ and a spectral bandwidth of 5.1 nm or up-chirped DS with a pulse energy of 5.1 nJ and an optical bandwidth of 9.6 nm can be generated. Similar polarization features are observed when the laser switches between different soliton operations as both CS and DS are group-velocity-locked vector solitons. Our work paves a novel way to generate dissipative solitons with a relatively high pulse energy (one order of magnitude larger than for CS) and a large chirp directly from an all-fiber net-anomalous-dispersion cavity through birefringent filter management.

## Introduction

Passive mode-locking in fiber lasers has been studied extensively over the past decades^1–7^. Mode-locked fiber lasers, as complex nonlinear dissipative systems, offer a versatile and promising platform for the study of soliton dynamics. Various types of solitons, including conventional solitons^2,3^, dispersion-managed solitons^4^, amplifier similaritons^5^ and dissipative solitons^6,7^, have been generated from ultrafast fiber lasers with different cavity parameters. It is well known that dispersion plays an important role in the soliton dynamics and that the associated optical pulse spectrum can show different characteristic features based on dispersion regime. In the context of this manuscript, the terminology ‘conventional solitons’ refers to solitons with characteristic Kelly sidebands which are usually generated in all-anomalous or net-anomalous dispersion regime due to the balance of anomalous dispersion and nonlinearity^2,3^. While all solitons generated from laser cavities undergo a characteristic pulse shaping process influenced by nonlinear gain and loss, dissipative solitons are usually associated with pulses experiencing significant energy dissipation in the cavity. They are typically up-chirped solitons with higher pulse energy generated in an all-normal or net-normal dispersion regime. The formation of dissipative solitons requires an intricate and mutual interaction of gain, loss, dispersion, and nonlinearity^6,7^.

Another important parameter for the generation of solitons is the fiber birefringence. Depending on the fiber birefringence and polarization-asymmetry in the laser cavity, different types of solitons can be generated, including scalar solitons^8^, group-velocity-locked vector solitons^9–12^, polarization-rotation vector solitons^13^ and polarization-locked vector solitons^14^. Both group-velocity-locked vector conventional solitons (GVLVCS)^9,10^ and group-velocity-locked vector dissipative solitons (GVLVDS)^11,12^ have been reported in fiber lasers. For group-velocity-locked vector solitons, a wavelength difference typically exists between orthogonal polarization components to balance the group-velocity difference induced by fiber birefringence which enables soliton trapping^9^. Also, by recombining the projections of phase-delayed rotated group-velocity-locked vector solitons, polarization-manipulated vector solitons with complex temporal and spectral structures resembling those of higher-order vector solitons can be constructed from both GVLVCS^10^ in an anomalous-dispersion regime and GVLVDS^12^ in a normal-dispersion regime.

It has been shown that spectral filtering effects can shape a pulse and induce the transition between different mode-locking regimes^15–18^. Switching between amplifier similaritons and dissipative solitons has been experimentally demonstrated in one single laser oscillator with an adjustable intracavity spectral filter^15^. For a net-anomalous-dispersion cavity, up-chirped solitons are numerically predicted by inserting a spectral filter into the cavity^16^. Conventional solitons and dissipative solitons with similar pulse energy of tens of pJ have been generated in cavities with fixed net-normal dispersion that include a piece of polarization-maintaining fiber (PMF)^19,20^. However, the generation of both chirp-free CS and up-chirped DS in a cavity with a fixed net-anomalous dispersion has not been demonstrated experimentally so far. The ability to generate highly-chirped and compressible dissipative soliton pulses in a net-anomalous cavity will be attractive for seeding chirped pulse amplification systems at wavelengths where normal dispersion components are not easily available.

In this letter, we propose an all-fiber thulium-doped fiber ring laser which can support two types of nonlinear waves in the anomalous-dispersion regime. Instead of tailoring the overall cavity dispersion to the normal regime, birefringent filter management is introduced to support higher pulse energies. By adjusting the polarization controller settings and the pump power, either transform-limited conventional solitons or up-chirped dissipative solitons can be obtained at the laser output. The pulses are group-velocity-locked vector solitons with similar polarization features. An intracavity Lyot filter with an adjustable extinction ratio enables the transition between different mode-locking regimes by altering the pulse dynamics. Dual-wavelength dissipative soliton mode-locking is also observed through birefringent filter management in the same cavity. This laser cavity offers a novel way to achieve dissipative solitons with a high pulse energy and a broad spectrum in the anomalous-dispersion regime without being limited by the area theorem for conventional solitons for the first time, to the best of our knowledge. These results can also provide deeper insights into the polarization states of different ultrashort pulses generated in the same cavity and expand the operating regime for single pulse formation in fiber lasers.

## Results

### Experimental setup

**Figure 1:**
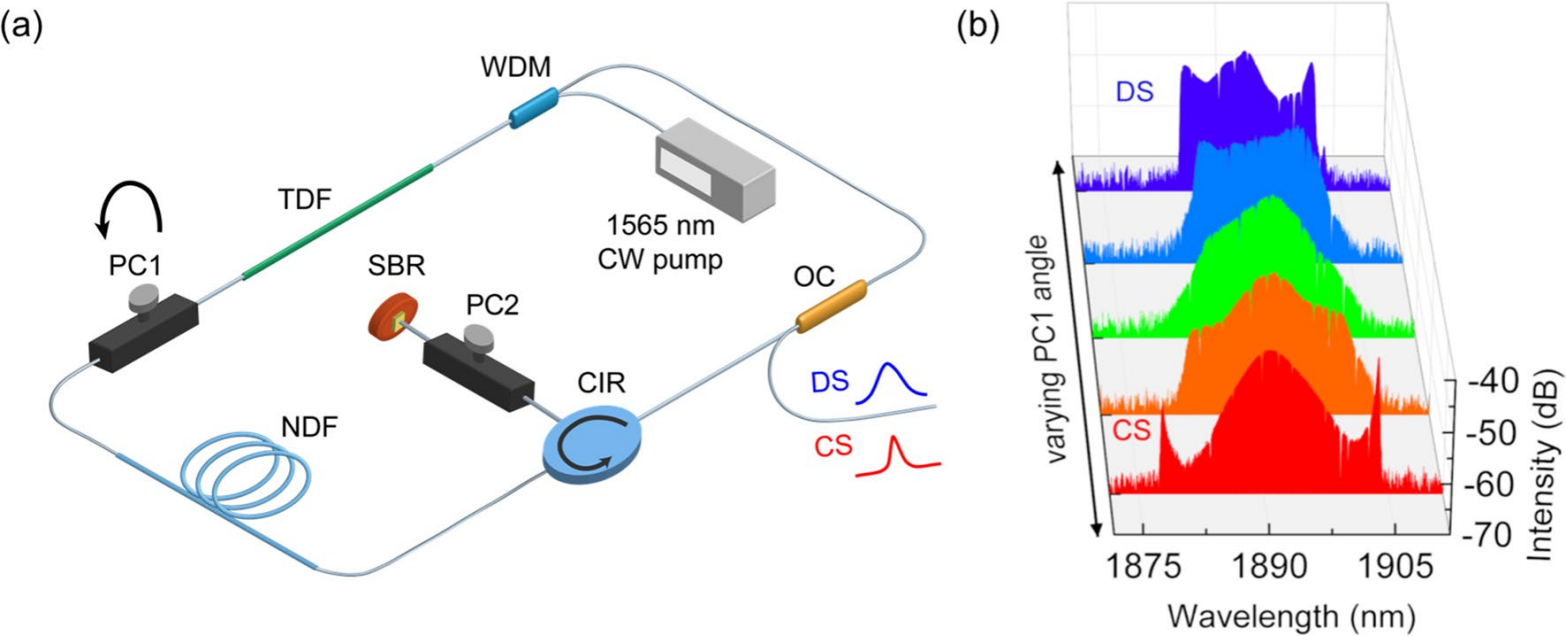
(**a**) Schematic diagram of the Tm fiber laser cavity. Depending on the polarization controller PC1 setting, either chirp-free conventional solitons (red) or up-chirped dissipative solitons (blue) can be obtained at the output. (**b**) Spectral evolution of the mode-locking states from CS (red) to DS (blue) with varying PC1 angle.

Figure 1 shows the schematic diagram of our all-fiber laser ring cavity. A 1.6-m long segment of thulium-doped gain fiber (TDF) (Coractive, TH512) is backward pumped by a 1565 nm continuous-wave (CW) laser through a wavelength-division multiplexer (WDM)(Thorlabs). Self-starting mode-locking is initiated by a saturable Bragg reflector (SBR)(Batop), which is butt-coupled to one port of the fiber circulator (CIR)(Precision micro optics). The circulator enforces unidirectional light propagation in the cavity. An 8.3-m long segment of normal dispersion fiber (NDF) (Nufern, UHNA4) is placed between the circulator and the gain fiber to construct a dispersion-managed cavity. The laser output is coupled from the 75% port of a fiber output coupler (OC)(Thorlabs). Two inline polarization controllers (PC) are placed in the cavity to optimize the mode-locking and to induce different states. All the other passive fibers are standard single mode fibers (SMFs). The group velocity dispersion of the gain fiber, the normal dispersion fiber and the single mode fiber are evaluated at − 73 ps^2^/km, 93 ps^2^/km and − 71 ps^2^/km, respectively^21^. Dispersion management is achieved by adjusting the length of the single mode fiber in the cavity. Thus, the total cavity length is adjustable between 26.9 and 16.9 m, resulting in an estimated net cavity dispersion from -0.55 to 0.16 ps^2^, covering a large range in both a net-anomalous and net-normal dispersion regime. A fiber polarization beam splitter (PBS) (DKphotonics), in combination with an external polarization controller, is used to analyze or manipulate the polarization state of the output pulses.

### Conventional and dissipative soliton mode-locking

By carefully optimizing the length of the single mode fiber, the net-dispersion is estimated around -0.2 ps^2^, which enables studying the mode-locking performance of the cavity in an anomalous-dispersion regime. Several mode-locking states with different spectral structures and two distinct types of stable solitons (conventional solitons and dissipative solitons) are generated from the same cavity. A representative spectral evolution from CS to DS, captured by an optical spectrum analyzer (OSA), is depicted in Fig. 1b. For a fixed pump power, this transition can be enabled by rotating one of the intracavity polarization controllers (PC1). Starting from a multi-pulsing conventional soliton state, the laser output transits into a single-pulsing dissipative soliton mode-locking state after passing through several unstable intermediate states with various spectral shapes. The center wavelength remains stable while the mode-locking regime evolves. The net cavity dispersion experienced by both CS and DS pulses is very similar since they share the same fiber path. Thus, the switching dynamics between the two states cannot be explained by a significant change in the net dispersion.

**Figure 2:**
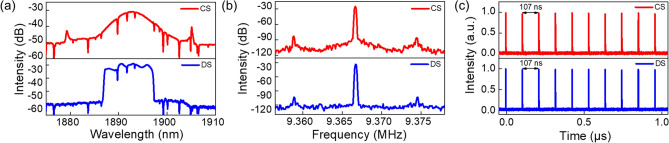
(**a**) Optical spectra, (**b**) RF spectra, and (**c**) oscilloscope traces of conventional solitons (red) and dissipative solitons (blue).

Self-starting mode-locking in the conventional soliton regime can be obtained when the intracavity PCs are carefully adjusted for pump power values above 335 mW coupled into the WDM. The average output power is 3.8 mW. The optical spectrum has a typical sech^2^ shape with a center wavelength of 1893 nm and a 3-dB bandwidth of 5.1 nm, see Fig. 2a. A pair of symmetric Kelly sidebands, a typical characteristic of conventional solitons, is visible in the optical spectrum with a separation of 25.7 nm. The net cavity dispersion derived from the position of Kelly sidebands is -0.27 ps^2^^22^, which is close to the value estimated from the fiber dispersion of − 0.2 ps^2^. Figure 2b shows the radio frequency (RF) spectrum of the conventional solitons (measured with a resolution bandwidth of 100 Hz). The fundamental repetition rate is 9.37 MHz and the RF signal has a signal-to-noise ratio (SNR) of ~ 58 dB, indicating stable mode-locking. The output pulse energy is 406 pJ. Due to relaxation oscillations^23^, two small sidebands exist in the RF spectrum with an offset of 7.8 kHz from the fundamental frequency, as frequently observed in Tm-doped fiber lasers. Figure 2c represents a typical mode-locked pulse train measured by a 20-GHz digital oscilloscope together with a 12.5-GHz InGaAs photodetector. The pulse spacing of 107 ns is consistent with the cavity roundtrip time and confirms stable single-pulsing mode-locking.

When the pump power is further increased, multi-pulsing occurs, as expected, for conventional solitons due to the peak-power-clamping effect^24^. By carefully rotating the intracavity polarization controller PC1, single-pulsing dissipative solitons can be generated with an average output power of 47.7 mW and an output pulse energy of 5.1 nJ (for a launched pump power of 529 mW), which is one order of magnitude higher than that of the conventional solitons. Such pulse energies are among the highest reported in dissipative solitons generated from single-mode Tm-doped fiber laser cavities, which typically have a pulse energy from 0.5 to 5 nJ^7,25–28^. As shown in Fig. 2a, the spectrum of this state features steep edges, which are characteristic for dissipative solitons^6^. The spectrum is centered at the wavelength of 1893 nm with an edge-to-edge bandwidth of 9.6 nm. Figure 2b shows the RF spectrum of the dissipative solitons. The repetition rate of 9.37 MHz is the same as that of the conventional solitons, while the SNR increases to ~ 70 dB. The oscilloscope trace shows pulses separated by a roundtrip time of 107 ns, confirming single-pulsing operation, see Fig. 2c.

### Polarization characterization and manipulation

We further investigated the polarization states in both mode-locking regimes. Both CS and DS exhibit a vector soliton nature due to the intrinsic fiber birefringence. With the external polarization controller and the fiber PBS, we can either separate their fundamental orthogonal polarization modes or generate polarization-manipulated vector solitons.

**Figure 3:**
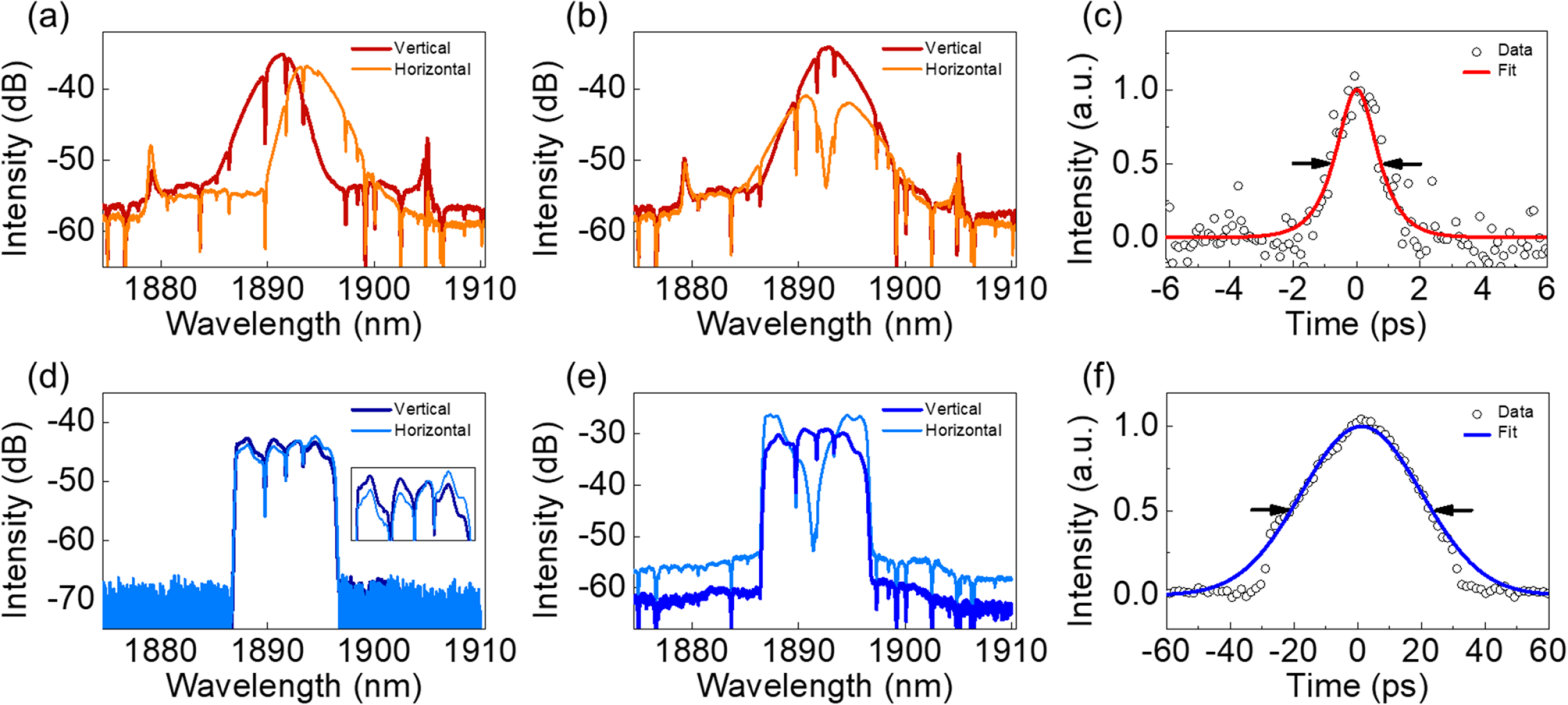
(**a**) Polarization-resolved spectra of the fundamental modes of GVLVCS. (**b**) Spectra of the polarization-manipulated GVLVCS. (**c**) AC trace and sech^2^ fitting of the vertical component in (**b**). (**d**) Polarization-resolved spectra of the fundamental modes of GVLVDS. Inset: zoom-in of the spectra. (**e**) Spectra of polarization-manipulated GVLVDS. (**f**) AC trace and Gaussian fitting of the vertical component in (**e**).

For the conventional solitons, by carefully adjusting the external PC, the two orthogonal fundamental modes of the vector solitons can be aligned to the vertical and horizontal axes of the PBS and resolved separately. The OSA spectra along the two polarization axes after the PBS are shown in Fig. 3a. The spectra of the vertical and horizontal components have slightly different center wavelengths of 1891.0 nm and 1893.8 nm, respectively. The difference in the center wavelengths of the orthogonal polarization components is a typical feature of group-velocity-locked vector solitons^9^. The two coupled solitons experience a center wavelength shift through self-phase and cross-phase modulation to compensate for the group-velocity difference caused by fiber birefringence. The pulse duration, measured by an intensity autocorrelator, is 1.3 ps for both orthogonal components, if a hyperbolic secant pulse shape is assumed. For both polarization components, there are no polarization sidebands in the RF spectra nor any amplitude modulation in the oscilloscope traces, confirming the GVLVCS nature.

With appropriate adjustment of the external PC, the polarization state can be rotated and an additional temporal phase delay can be induced between the orthogonal components so that the combination of their projections on the PBS forms complex pulse structures in form of polarization-manipulated vector solitons. In this case, a spectral dip appears along the horizontal axis. The position and depth of the spectral dip is dependent on the rotation and the temporal phase difference induced by the PC and we record the spectra of both axes when the dip reaches its most pronounced depth at the center of the spectrum, as shown in Fig. 3b. Such a spectral modulation is caused by the interference between the projections of the two phase-delayed orthogonal polarization components on the PBS^12^. The interference between the projections leads to a double-hump temporal structure which resembles that of a higher-order vector soliton. For the vertical axis, no spectral dip is present, and the spectrum has a narrower bandwidth of 3.8 nm compared to that before the PBS. Assuming a hyperbolic secant shape, the pulse duration is 1 ps, see the autocorrelation (AC) trace in Fig. 3c. The calculated time-bandwidth product (TBP) is 0.317, which is very close to the transform limit value of 0.315.

Polarization-resolved measurements for the dissipative solitons in Fig. 3 reveal that the DS is also group-velocity-locked. Depending on the external PC setting, as shown in Fig. 3d,e, either fundamental polarization modes of the vector dissipative solitons with slightly different center wavelengths (vertical: 1891.4 nm, horizontal: 1892.0 nm) or polarization-manipulated vector dissipative solitons can be obtained after the PBS. For the fundamental polarization modes, the pulse duration is 39.3 ps and 41.1 ps for the vertical and horizontal components, respectively, if a Gaussian shape is assumed. For the case of polarization-manipulated dissipative vector solitons, similar to that of conventional solitons, an interference-induced spectral dip appears at the center of the spectrum along the horizontal axis. At the same time, a narrower spectrum with a 3-dB width of 7.6 nm is observed along the vertical axis. By fitting the AC trace along the vertical axis in Fig. 3f with a Gaussian function, the pulse duration is calculated to be 31.5 ps. The slight clipping of the AC trace is caused by the limited range of our autocorrelator. However, it is confirmed that the clipping has negligible impact on pulse duration measurement by checking a pulse with known duration. Different from the close to chirp-free conventional soliton, the DS pulse is highly up-chirped with a TBP of 20.0 and can be compressed externally with propagation in a single mode fiber with anomalous-dispersion. As before for the CS, the RF spectra do not feature polarization sidebands and the pulse trains show no amplitude modulation, which agrees well with the GVLVDS nature.

## Discussion and conclusion

For the first time, two types of coherent group-velocity-locked vector solitons are generated in one single laser cavity. Although the PC-induced stress may change the local fiber birefringence, it has little impact on the overall cavity birefringence^29^. Thus, we find that soliton trapping can occur for both CS and DS with similar net cavity birefringence values. Compared to the group-velocity-locked vector conventional solitons with a wavelength shift of 2.7 nm between orthogonal polarization modes, the group-velocity-locked vector dissipative solitons exhibit a smaller wavelength offset of 0.6 nm. Since the peak power of the dissipative solitons (152 W) is lower than that of the conventional solitons (361 W), a smaller frequency shift is needed to compensate for the birefringence-induced group-velocity difference due to the decreased nonlinear birefringence in the cavity. This is consistent with previous findings of noise-like pulse trapping with a large wavelength shift caused by high peak powers^30^.

The formation mechanism of both soliton states may be explained by a spectral filtering effect originating from a Lyot filter. Due to the polarization-dependent loss of components like the WDM, OC, and CIR, a periodic Lyot filter caused by fiber birefringence can be formed in the cavity, even without the presence of an intracavity polarizer^31^. By characterizing the individual transmission characteristics of all laser components including the CIR, OC, WDM and SBR, we verified that no other periodic filter exists in our cavity as the combined transmission spectrum of these components in our cavity has only one single peak with a 3-dB bandwidth of more than 70 nm. Our cavity featuring a polarization-asymmetry can support vector soliton operation since vector solitons can even be generated in strongly polarization-dependent cavities^32^.

**Figure 4:**
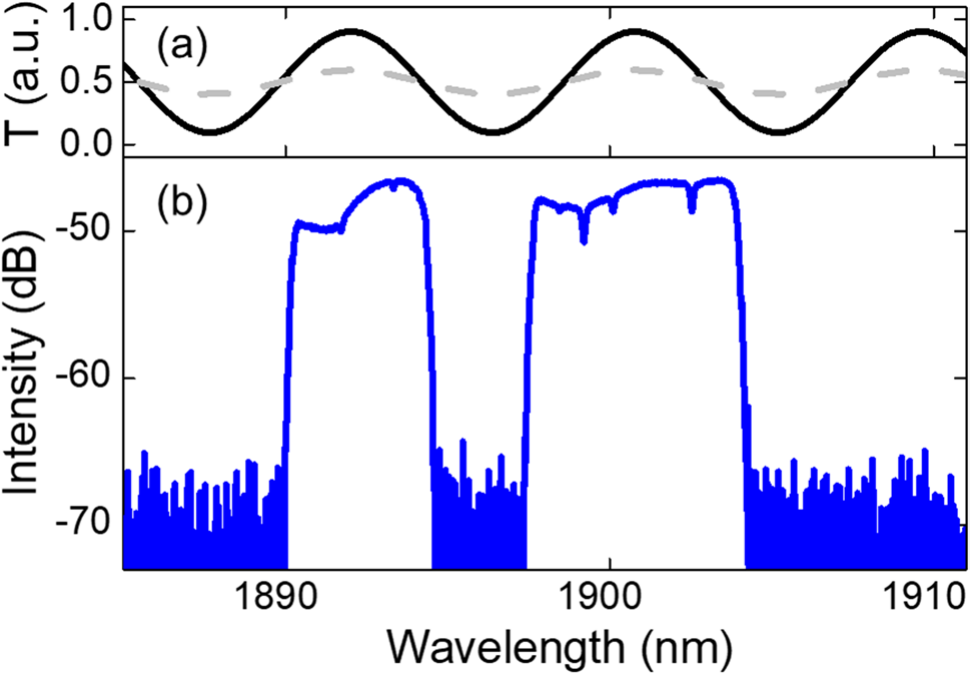
Dual-wavelength dissipative soliton mode-locking induced by the Lyot filter. (**a**) Simulated Lyot filter transmission spectra for two different intracavity polarization controller angles, showing different extinction ratios. (**b**) Spectrum of the dual-wavelength dissipative solitons.

Such a spectral Lyot filter can shape a pulse in several ways: Apart from shortening a chirped pulse, it can affect the temporal phase and thus the chirp of a pulse by adding an opposite chirp to the input pulse. This provides a pathway for pulses in an anomalous-dispersion regime to accumulate a chirp with opposite sign during pulse build-up and converge to up-chirped solitons^16^. In our cavity, the period of the Lyot filter is mainly determined by the overall fiber birefringence. However, the Lyot filter can have different extinction ratios by adjusting the intracavity polarization controllers^29,33^. As shown in Fig. 4a, the modulation depth of the simulated Lyot filter in our cavity can be tuned with different angles of the intracavity polarization controller. Thus, CS and DS states can evolve under different filtering conditions. Details of the Lyot filter can be found in the Methods section. Even with the dispersion management, the cavity is predominantly anomalous. When the modulation depth of the Lyot filter is relatively small after optimization of the polarization controller, its effect becomes negligible for the pulse shaping and conventional solitons with Kelly sidebands can be generated, as expected in a net-anomalous-dispersion cavity. It has been numerically predicted that the transition between mode-locking regimes can be highly sensitive to the bandwidth of an intracavity filter^18^. So, if a Lyot filter with a high extinction ratio is present in the cavity, the effective filter bandwidth becomes narrow enough to affect the pulse dynamics. In this case, the pulse experiences an up-chirp from the filter so that the cavity converges to a dissipative soliton operation. The self-consistency of the intracavity dissipative solitons is enforced by the filtering effect and the saturable absorber.

Dual-wavelength or multi-wavelength mode-locking operation can be obtained with a periodic filter like Lyot filter in the cavity^29,31,34,35^. As there is no other periodic filter present, the existence of such a birefringent Lyot filter is supported by our observation of dual-wavelength dissipative soliton mode-locking in the same cavity. The spectrum of the dual-wavelength mode-locking state is shown in Fig. 4b. The center wavelengths of the two dissipative solitons are found at 1892.3 nm and 1900.8 nm, respectively. The estimated transmission spectrum of the Lyot filter, presented as the solid line in Fig. 4a, overlaps well with the dual-wavelength lasing, supporting the experimental findings. The difference in the spectral bandwidths of the two dissipative solitons might be caused by the asymmetric net gain at the two wavelengths, leading to different mounts of spectral broadening induced by self-phase modulation, similar to other multi-wavelength dissipative solitons reported^34,35^. Both the dual-wavelength mode-locking state as well as the transition between mode-locking regimes are enabled by this Lyot filter, whose filtering periodicity is formed without any PMF in the cavity. Our configuration thus varies from the cavity design^20^ in which a piece of PMF is essential for the formation of two different types of solitons through mode-coupling between SMF and PMF. The presented transition between CS and DS states has been reproduced in two other cavities with a similar design but with different fiber lengths (corresponding to repetition rates of 7.8 MHz and 13.7 MHz). Thus, we could demonstrate that a general alignment procedure, consisting of the rotation of one intracavity polarization controller, can robustly induce such a transition once mode-locking has been achieved in a soliton regime. This supports further that such a tunable Lyot filter can be regarded as a universal mechanism inducing these mode-locking dynamics.

**Figure 5:**
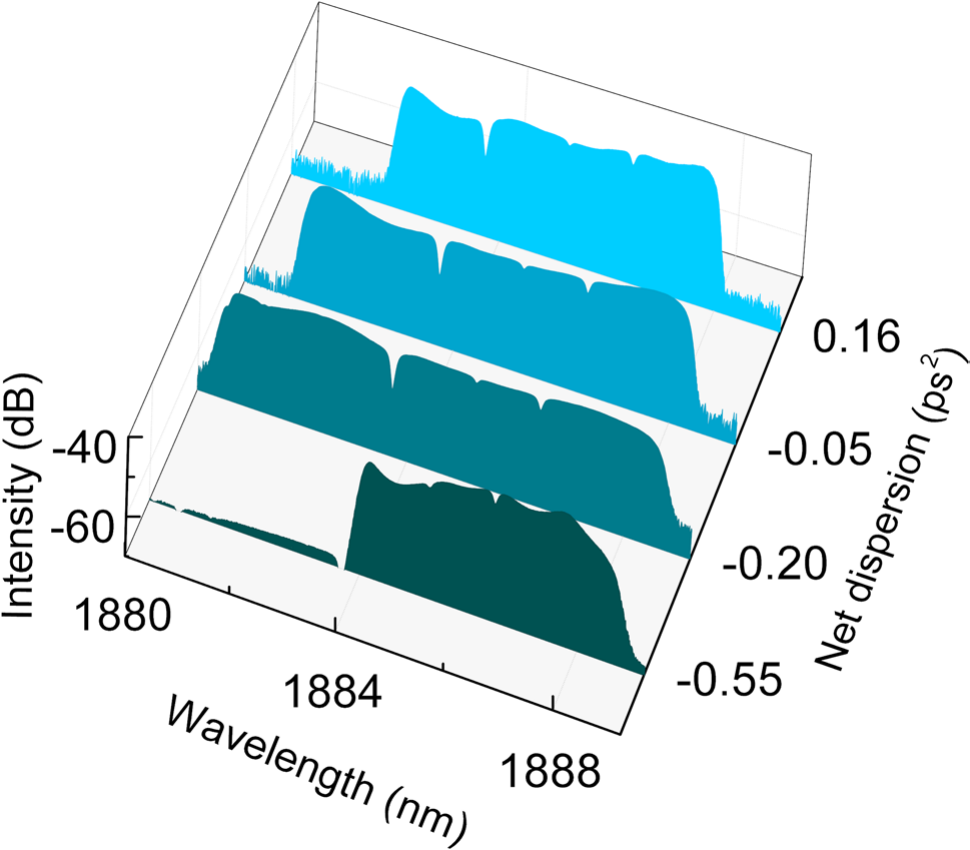
Spectra of dissipative solitons generated from four cavities with different net-dispersion values.

We further examined the generation of dissipative solitons in different dispersion regimes by varying the length of the single mode fiber in the cavity from 7 to 17 m. Stable dissipative solitons are obtained for various net-dispersion values, ranging from a large normal-dispersion regime (0.16 ps^2^) to an anomalous-dispersion regime with significant dispersion (-0.55 ps^2^). The spectra of the dissipative solitons obtained from the four cavities with different representative net-dispersion values are shown in Fig. 5. All the spectra exhibit steep edges and the pulses are highly-chirped, confirming the DS generation in all cases. Thus, we validate that the generation of dissipative solitons in the anomalous-dispersion regime of such cavity is a universal phenomenon. To the best of our knowledge, this is the first time that highly chirped DSs with characteristic spectra are generated in the net-normal, near-to-zero and net-anomalous dispersion regimes of a single cavity. It should be noticed that the dissipative solitons here are distinct from dissipative soliton resonance pulses as the peak power is not fixed while the pump power is varied^36^.

In conclusion, we demonstrate the generation of both group-velocity-locked vector conventional solitons and dissipative solitons from a single all-fiber thulium-doped laser with a fixed net dispersion although they typically exist in different dispersion regimes. For the first time, up-chirped dissipative solitons with high pulse energy are obtained experimentally in the same net-anomalous-dispersion cavity as chirp-free conventional solitons with lower pulse energy, resulting in a pulse energy boost by a factor of 12.6 and a 31.5-fold increase in pulse duration. Without changing the cavity configuration, the pulse dynamics can be controlled by managing the intrinsic spectral Lyot filter through an intracavity polarization controller. A filter-induced up-chirp enables the generation of dissipative solitons in the net-anomalous dispersion regime. The group-velocity-locked vector soliton nature is preserved when the laser switches between different soliton operations. Similar features induced by interference between polarization-manipulated projections are observed for both pulses. This finding increases our understanding of the evolution of polarization states during the transition between different mode-locking regimes. The results in this paper describe a new type of mode-locking dynamics which is distinct from previously reported phenomena. Our all-fiber oscillator paves a novel way to generate energetic ultrashort pulses in the net-anomalous dispersion regime. The ability to generate two types of ultrashort pulses with different characteristics could be further combined with automated mode-locking approaches^37^, making our ultrafast laser source attractive for applications benefiting from different pulse profiles.

## Methods

### Lyot filter

Based on a Jones matrix calculation^38^, the transmittance of the Lyot filter in our cavity can be expressed by the following formula:

1 $$T = \cos^{2} \theta_{1} \cos^{2} \theta_{2} + \sin^{2} \theta_{1} \sin^{2} \theta_{2} + \frac{1}{2}\sin \left( {2\theta_{1} } \right)\sin \left( {2\theta_{2} } \right)\cos \left( {\frac{2\pi L\Delta n}{\lambda }} \right)$$

Here, T is the transmittance of the Lyot filter, $\Delta n$ is the fiber birefringence, L is the length of cavity, $\lambda$ is the wavelength, $\theta_{1}$ is the angle between the polarization direction of the light and the fast axis of the birefringent fiber, and $\theta_{2}$ is the angle between the orientation of polarization-dependent component and the fast axis of the birefringent fiber. The free spectral range (FSR) of the Lyot filter is inversely proportional to the total birefringence in the cavity:

2 $${\text{FSR}} = \frac{{\lambda^{2} }}{L\Delta n}$$

The fiber birefringence in the cavity is estimated to be $\sim 1.87 \times 10^{ - 5}$ based on our experimental observation of the dual-wavelength dissipative soliton mode-locking. With a cavity length of 21.9 m, the corresponding FSR is 8.75 nm, which matches well with the spacing of the dual-wavelength dissipative solitons of 8.5 nm. In the simulation, $\theta_{2}$ is assumed to be $\pi /4$ to study the tunable extinction ratio of Lyot filter qualitatively without loss of generality^38^. $\theta_{1}$ can be changed by the rotation of the intracavity polarization controllers. By setting $\theta_{1}$ to different values, Lyot filters with different extinction ratios can be obtained. In Fig. 4a, a strong Lyot filter with a high extinction ratio is obtained when $\theta_{1}$ is set to $13\pi /20$ (black solid curve) while a Lyot filter with relatively low extinction ratio is generated with $\theta_{1}$ value of $17\pi /32$ (gray dotted line).
